# Understanding Patient Rights: A Pilot Study Assessing Health Literacy in Written Pre-Appointment Letters

**DOI:** 10.3390/ijerph22101518

**Published:** 2025-10-03

**Authors:** Julie Dalgaard Guldager, Lotte Christina Waldhauer, Carsten Kronborg Bak

**Affiliations:** 1 Research Department, University College South Denmark, 6705 Esbjerg, Denmark; 2 Research Program for Social Work, Administration and Communication, University College South Denmark, 6705 Esbjerg, Denmark; lcwa@ucsyd.dk; 3 Health Department, University College Lillebælt, 5230 Odense M, Denmark; ckba56@outlook.dk

**Keywords:** health literacy, healthcare letters, patient rights, patient communication, digital literacy

## Abstract

This pilot study examined how sociodemographic factors (age, education, internet usage) influence patients’ comprehension of written healthcare communications, and their understanding of patient rights as articulated in appointment letters. A cross-sectional study was conducted among in-clinic patients at three Danish hospitals. Participants completed a self-administered questionnaire, assessing health literacy through four domains: assessing, understanding, appraising, and applying information from appointment letters. The questionnaire included sociodemographic data, Internet usage, IT competencies, and self-assessed health. Overall, 364 patients participated, with the majority being female and aged between 35 and 74 years. The mean scores for the domains of understanding and applying information were higher compared to assessing and appraising. Multiple linear regression analysis revealed that higher education levels positively correlated with the ability to appraise legal information, while frequent internet usage also enhanced appraisal skills. Findings highlight a concerning gap in patients’ ability to understand and appraise their patient rights within written healthcare communications. While patients demonstrate reasonable skills in understanding basic information, critical legal aspects remain challenging. Enhancing education and digital literacy may improve comprehension, emphasizing the need for simplified language and alternative formats in appointment letters. Further research is warranted to optimize communication strategies for patient rights.

## 1. Introduction

Health literacy is increasingly recognized as a key determinant of health outcomes, particularly as healthcare systems depend more on written communications [1,2]. Health literacy, often defined as an individual’s capacity to access, process, and comprehend basic health information and services, plays a pivotal role in patient engagement and empowerment in managing health [3] as well as in how individuals navigate healthcare systems and comprehend documents like pre-appointment letters.

In Denmark, pre-appointment letters serve as a critical aspect of healthcare interactions, preceding the initial face-to-face appointment. These written materials provide an important role in giving patients essential information about upcoming appointments, their medical care, and their patient rights, such as the right to receive a diagnosis within 30 days of referral [4] (For English-language patient information on the 30-day diagnostic right, see [5,6,7]).

The written communication in these pre-appointment letters also reflects the broader trends in digitalization and efficiency enhancement [8]. However, these letters often contain complex, technical language that may challenge individuals with lower levels of health literacy, potentially hindering their ability to understand and act on this important information [9] (for an example of the content of a Danish pre-appointment letter, see Supplementary Material S1). Recent evaluations continue to show that patient-facing materials (including digital/printable content) frequently exceed recommended readability levels, limiting comprehension and actionability [10,11].

Despite their potential contribution to patient health, rights, and navigation of the healthcare system, pre-appointed letters remains inadequately understood [12]. Research in current pre-appointment written materials is limited, both in terms of how they inform and prepare patients before appointments and their influence on the quality of care and patient well-being [13]. Moreover, as health systems shift tasks to patients via portals and digital letters, the comprehension burden increasingly falls on patients, heightening the importance of evaluating what people actually understand and can do with this information [14].

Studies consistently show that higher health literacy correlates with better health outcomes. Patients with stronger literacy skills are likelier to adhere to prescribed treatments, engage in preventive health measures, and navigate the healthcare system effectively [3,15,16]. In contrast, individuals with limited health literacy often struggle to understand written and oral health information, which can lead to adverse outcomes such as higher hospitalization rates and poorer overall health [17,18]. Research has thus shown that various interventions may improve health literacy among patients with chronic diseases [19].

Prior research highlights additional challenges, that health information is often overly complex, and laden with technical or medical jargon. Some materials contain inaccuracies and information gaps, particularly in failing to provide a balanced overview of the risks and benefits of, e.g., drug treatments. Such issues can lead individuals with low health literacy to either avoid taking medications or discontinue treatment prematurely [10,14].

However, a specific area of health literacy that remains under-researched is patients’ understanding of their rights as outlined in healthcare communications [14]. Despite the centrality of such rights to patient autonomy, many individuals may not fully understand the information about their rights due to the complex language used in pre-appointment letters [17]. This underscores the significant relationship between health literacy and patient empowerment, which is critical for effective healthcare navigation [2,15].

Why has this remained underexplored? First, disciplinary silos have treated patient rights primarily as a matter for law and policy rather than as a communication problem. Health-communication agendas have therefore concentrated on clinical instructions and risk/benefit discussions, while rights comprehension sat in legal scholarship. Landmark work on the “legal determinants of health” underscores law’s central role in shaping health, yet it is rarely operationalized as communication content to be measured for understandability [20].

Second, there is a measurement lock-in effect. Widely used health- and eHealth-literacy instruments emphasize functional skills (prose, numeracy, navigation) and seldom capture critical capacities such as recognizing, appraising, and asserting one’s legal entitlements. Recent systematic reviews of health-literacy and eHealth-literacy measures highlight these emphases and call for stronger coverage of critical dimensions [14].

This gap has material consequences. Across multiple settings, recent studies report uneven awareness and communication of patient rights (e.g., information rights, consent, and rights charters), indicating persistent discrepancies between formal legal guarantees and what patients take away from everyday communications [21]. Given Denmark’s statutory 30-day diagnostic right, insufficient comprehension can directly affect patients’ ability to exercise extended free-choice provisions if timelines slip [5,6].

A study by Rudd et al. [14] revealed that many health-related documents are written at a reading level significantly higher than the average patient’s comprehension level. This mismatch between the readability of healthcare documents and patients’ health literacy levels is a well-documented barrier to effective communication. However, research into patients’ understanding of the legal aspects of these communications, such as patient rights, remains limited. For instance, while many patients may understand logistical information about their appointments, they may not be fully aware of their right to receive a diagnosis within a certain time frame [2], which can have profound implications for their healthcare experience.

Moreover, the role of digital literacy in health communication is becoming increasingly important, as healthcare systems adopt more digital tools for patient interaction. Studies such as those by Norman & Skinner [22] indicate that digital literacy may enhance patients’ ability to navigate online health resources. As digital communication becomes more prevalent, understanding its impact on health literacy is essential for effective public health messaging [23]. Yet, it is unclear how this affects their understanding of written and legal health information. Additionally, while education and frequent Internet use have been linked to better health literacy [24], the specific impact of these factors on the comprehension of legal patient rights remains an under-explored area.

Recent reviews of eHealth-literacy instruments confirm rapid proliferation of tools but also note gaps in measuring appraisal/action skills needed to interpret and assert rights in digital environments [14,25].

The existing literature provides a comprehensive understanding of functional health literacy [26] and how patients comprehend medical instructions and follow treatment plans. However, it falls short in addressing how well patients understand their patient rights as conveyed in written healthcare communications. Research also shows that hospital staff perceptions of patients’ health literacy can significantly influence patient care and communication strategies [27]. A systematic review indicates that targeted health literacy interventions can improve patient outcomes [15]. Older adults often struggle with understanding prescription drug labels due to low health literacy levels, which can lead to adverse outcomes [17]. Taken together, these patterns justify a focused examination of how pre-appointment letters communicate statutory rights (e.g., Denmark’s 30-day diagnostic entitlement), and whether patients can recognize, interpret, and act on those rights in practice.

Given the growing emphasis on patient autonomy and informed decision-making, it is critical to address this gap. This study’s aim was: To investigate the influence of sociodemographic factors, including age, education, and internet usage, on patients’ comprehension of written healthcare communications, particularly focusing on their understanding of legal patient rights as articulated in appointment letters.

This pilot study explores patients’ comprehension of written healthcare communications. Addressing this research question, the study seeks to contribute to a more nuanced understanding of health literacy, particularly patient rights and written healthcare information. Digital access and IT competence were included as contextual predictors, and not as the primary outcome.

## 2. Materials and Methods

### 2.1. Study Design and Sampling

This cross-sectional study employed a quantitative survey-based methodology among in-clinic patients at three Danish hospitals. Data was collected at three departments over two to four months in 2023 and 2024. We purposively sampled three outpatient departments, Orthopedics, Gynecology, and Hematology to capture variation in clinical context and communication demands. Orthopedics represents high-volume elective/surgical care with brief encounters; Gynecology is a women-only service where rights information often intersects with sensitive, preference-dependent decisions; Hematology involves chronic and complex medical care with frequent written communication. Including a women-only department was intentional, as communication about patient rights in women’s health is both highly relevant and potentially distinctive.

A medical secretary distributed the anonymous self-administered questionnaire to patients. The questionnaire was introduced to participants, explaining that it could be completed either electronically via a QR code or in paper format by manually ticking the boxes. Only one respondent chose the electronic version.

### 2.2. Measures/Instrumentation

We explored the association of health literacy with participants’ sociodemographic characteristics, Internet usage, IT competence, and self-assessed health. The research team generated all research questions as closed-ended items, specifically for this study.

We conducted a pre-test of the questionnaire with six patients to assess feasibility (clarity, flow, and comprehensibility) prior to the main study rather than to conduct full psychometric validation. This process allowed us to identify and address issues related to the questionnaire’s structure and wording, as well as to verify the respondents’ understanding of the questions. Feedback from these telephone interviews was instrumental in making the necessary adjustments before the questionnaire was distributed in the hospital departments.

As an exploratory pilot study, we did not perform a formal sample size calculation; instead, we aimed to recruit as many eligible patients as possible during the study period to obtain preliminary data.

Rather than collapsing the measures into a single score, we examined each domain independently to identify domain-specific strengths and challenges (e.g., whether patients found it easier to understand basic information than to appraise legal details). This approach can reveal more nuanced associations; for instance, digital literacy might strongly relate to appraising information but less so to basic understanding.

The final questionnaire covered the following areas:

#### 2.2.1. Health Literacy

Questions used to establish health literacy in written appointment letters were inspired by the HLS-EU Consortium for measuring health literacy in the general population. Detailed information on the development and pre-testing is described in other publications [3,28]. The translation into the Danish version followed a standardized procedure completed by Sørensen, Maindal, and colleagues (unpublished material; please contact the third author, K. Sørensen, for further information).

**Figure 1:**
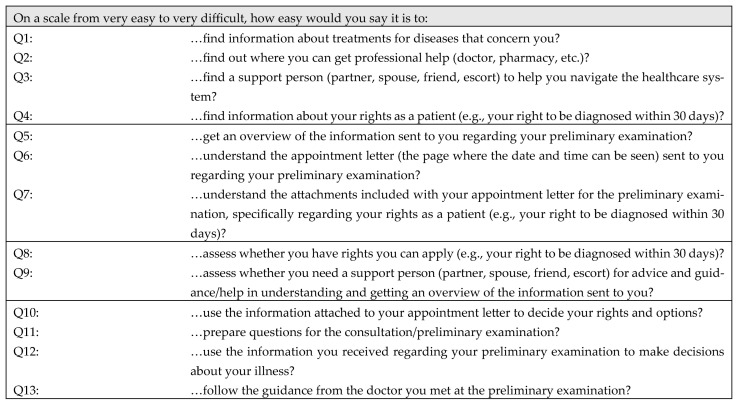
Questions used to establish health literacy in written appointment letters.

The survey was specifically designed for this pilot study. We developed a 13-item health literacy questionnaire specifically targeting comprehension of appointment letter content and patient rights, structured around four domains (assessing, understanding, appraising, and applying) derived from the Sørensen et al. health literacy framework (see Figure 1; for the Danish version see Figure S1 in the Supplementary Material S2). Each domain’s items were designed to reflect a stage in processing written health information. For instance, the assessing domain (4 items) asked how easily a patient could find and identify important details in the letter; the understanding domain (3 items) gauged how easily the patient understands the content; appraising (2 items) covered judging the trustworthiness or relevance of the information (including legal rights); and applying (4 items) addressed using the information in one’s healthcare decisions. The four questions designed to assess the patients’ ability to apply information were structured such that two questions focused on the informational material provided before the preliminary examination. In comparison, the remaining two questions addressed the application of information after the initial examination.

The domain of assessing written appointment letters was established by a sum score of questions 1 to 4 (Q1–Q4) (Cronbach’s alpha 0.779), the domain of understanding from Q5–Q7 (Cronbach’s alpha 0.840), the domain of appraising from Q8–Q9 (Cronbach’s alpha 0.748), and the domain of applying from Q10–Q13 (Cronbach’s alpha 0.856). A composite score of health literacy was calculated by summing the four domains.

To strengthen validity, the questionnaire development process incorporated several steps beyond expert review. Content validity was ensured by adapting items from the validated HLS-EU framework and having them assessed by health communication experts. Face validity was established through pre-testing with patients, which confirmed comprehensibility and relevance of the items in a clinical setting. Internal consistency for the domains was acceptable, as indicated by Cronbach’s alpha values. Health communication experts reviewed this questionnaire for content validity, but it has not yet undergone extensive psychometric validation. The present pilot study serves as an initial step in validating this tool.

All questions could be answered on a 4-point Likert scale (very easy, easy, difficult, very difficult) with the option to respond, “don’t know”. In line with the HLS-EU study [28], all “don’t know” responses were treated as missing values, as they cannot be interpreted as valid answers and may reflect uncertainty or disengagement. This approach reduces potential bias in scale scores and ensures comparability with previous applications of the instrument. Results were recoded for the sum score of the four domains so that higher values reflected higher health literacy.

As highlighted in the research aim, we included questions about patients’ understanding of their legal rights in written appointments across the four HL dimensions (see Figure S2 in Supplementary Material S3). The figure illustrates more precisely how we included the questions in different phases of the information process.

#### 2.2.2. Sociodemographic Characteristics

Participants were asked about their gender (male or female) and age. Participants could choose between age groups covering ten years (16–75+), which were further divided into four categories (16–34, 35–54, 55–74, and 75+ years) for statistical analysis. Participants were asked to indicate their civil status (married, in a partnership, divorced, widowed, or unmarried). For statistical analysis, three categories were created: married/partnership, divorced/widowed, and unmarried. Education level was established based on the highest completed education level: primary school, secondary school, short-cycle higher education, bachelor’s degree, master’s degree or higher, and “other”. For statistical analysis, primary and secondary schools were grouped, and students and “other” were combined. To establish occupational status, participants were asked to indicate if they were employed, unemployed, early retiree, retiree, senior citizen, student, or “other”. For statistical analysis, all categories other than employed were grouped as “not employed”.

#### 2.2.3. Internet Usage and IT Competence

Participants were asked, “How often do you use the Internet?”. Responses were recorded on a 4-point Likert scale (almost every day, several times per week, around one time per week, or rarely). The first two categories were grouped as “frequent usage”, and the last two as “rare or absent usage”. Further, participants were asked, “How do you assess your IT competence?”. Responses were recorded on a 5-point Likert scale (excellent, good, average, fair, poor). The first three categories were grouped as good IT competence, and the last two as poor IT competence. Digital access and IT competence were included as contextual predictors; they were not primary outcome.

#### 2.2.4. Self-Assessed Health

Self-assessed health was measured with the question, “What is your assessment of your health?”. The five response categories were: “My health is excellent”, “My health is excellent”, “My health is good”, “My health is less good”, and “My health is bad”. For statistical analysis, the first three categories were grouped as good self-assessed health, while the remaining two were grouped as poor self-assessed health.

### 2.3. Statistical Analysis

Absolute and relative frequencies were used to describe the characteristics of the participants (Table 1). Mean values and standard deviations were used to describe the participants’ four domains of self-assessed health literacy competence (assessing, understanding, appraising, and applying), on a scale from 1 to 4, with higher values indicating higher health literacy (Table 2). Multiple linear regression analysis was used to determine the association between the included variables and the participants’ four domains of self-assessed health literacy competence (Table 3). This method was chosen to allow for simultaneous adjustment of multiple predictors and to reduce potential confounding.

Independent variables were selected a priori based on theoretical relevance and prior evidence (age, sex, education, occupational status, department, IT competence, and self-assessed health). Sex was included to adjust for the gender imbalance introduced by the inclusion of the gynecology department. We verified that the assumptions of multiple linear regression (linearity, normal distribution of residuals, and homoscedasticity) were satisfied for each model. Analyses were conducted on available cases for each variable, resulting in different denominators across participant characteristics and health literacy domains. Missing data, including “don’t know” responses, were treated as missing and handled by listwise deletion in regression models.

Results were presented as beta coefficients (β), 95% confidence intervals, and *p*-values. Analyses were conducted separately for each independent variable (crude model, Model 1, see Table S1 in Supplementary Material S4) and concurrently (adjusted model, Model 2, see Table 3). IBM SPSS Statistics, version 28 (Windows), was used for all analysis.

### 2.4. Ethical Approval

This study follows the principles outlined in the Declaration of Helsinki [29]. All participants received an information folder with the invitation to participate and were informed that their participation was voluntary.

## 3. Results

### 3.1. Participants Characteristics

A total of 364 patients participated in the survey. More women (75.3%) than men (24.7%) participated. The majority aged 35–54 years (34.6%) or 55–74 years (39.8%), and two-thirds (66%) were married or in a partnership. The educational level of the participants differed, with most holding a bachelor’s degree (38.1%) followed by those having finished short-cycle higher education (21.2%). Slightly more participants were employed (52.6%), the majority used the Internet often (95.9%), and the majority assessed their own IT competence (82%) and self-assessed health (73%) as good (see Table 1).

**Table 1:** Characteristics of participants.

	Frequency/Percent
**Sex** (*n* = 364)	
Female	274 (75.3)
Male	90 (24.7)
**Age group** (*n* = 364)	
16–34	59 (16.2)
35–54	126 (34.6)
55–74	145 (39.8)
>75	34 (9.3)
**Civil status** (*n* = 362)	
Married/partnership	239 (66.0)
Divorced/widowed	57 (15.7)
Unmarried	66 (18.2)
**Education** (*n* = 354)	
Primary/secondary education	47 (13.3)
Short-cycle higher education	75 (21.2)
Bachelor’s degree	135 (38.1)
Master’s degree or higher	45 (12.7)
Other	52 (14.7)
**Occupational status** (*n* = 361)	
Employed	190 (52.6)
Not employed	171 (47.4)
**Usage of Internet** (*n* = 362)	
Rare or absent	15 (4.1)
Frequent	347 (95.9)
**IT competence** (*n* = 361)	
Poor	65 (18.0)
Good	296 (82.0)
**Self-assessed health** (*n* = 256)	
Poor	69 (27.0)
Good	187 (73.0)

### 3.2. Summary of Findings

Table 2 shows the self-assessed health literacy overall and across the domains of assessing, understanding, appraising, and applying written appointment letters. The domain of understanding information (mean 3.250) followed by applying information (mean 3.144), were the highest-rated health literacy domains of participants, with assessing (mean 3.017) and appraising information (mean 3.070) following.

**Table 2:** Self-assessed health literacy domains * of assessing, understanding, appraising, and applying information from written appointment letters, along with health literacy totals, measured on a scale from 1 to 4 (*n* = 364).

	Mean	SD
**Accessing information** (*n* = 237)	3.017	0.569
**Understanding information** (*n* = 315)	3.250	0.593
**Appraising information** (*n* = 257)	3.070	0.668
**Applying information** (*n* = 213)	3.144	0.577
**Health literacy total** (*n* = 164)	3.158	0.513

* see Figure S2 in the Supplementary Materials for further illustration of the analytical framework.

The multiple linear regression analysis model (see Table 3) showed that having a short-cycle higher education (compared to primary/secondary education) (β = 0.264, 95% CI: 0.012–0.852) was positively associated with the health literacy domain of appraising written appointment letters. Similarly, using the Internet often (compared to rarely or never) (β = 0.201, 95% CI: 0.062–1.481) was also positively associated with the health literacy appraising domain. No statistically significant associations were found between the remaining variables in the appraising domain, nor for any variables in the domains of assessing, understanding, or applying written appointment letters. Likewise, no significant relationships were found between the background variables and the total health literacy score. Accordingly, participants’ overall health literacy was similar across different sociodemographic subgroups, indicating that age, gender, education level, and internet use did not markedly influence this sample’s combined health literacy measure in this sample.

**Table 3:** Multiple linear regressions for factors associated with self-assessed health literacy domains of assessing, understanding, appraising, and applying information from written appointment letters.

	Assessing		Understanding		Appraising		Applying		Health Literacy Total	
	Adjusted—Model 2		Adjusted—Model 2		Adjusted—Model 2		Adjusted—Model 2		Adjusted—Model 2	
	*n* = 237 *		*n* = 315 *		*n* = 257 *		*n* = 213 *		*n =* 164 *	
	β	95% CI	β	95% CI	β	95% CI	β	95% CI	β	95% CI
Sex										
Male (0)	Ref.		Ref.		Ref.		Ref.		Ref.	
Female (1)	0.099	(−0.091, 0.365)	0.092	(−0.070, 0.326)	−0.010	(−0.276, 0.245)	0.035	(−0.188, 0.279)	0.116	(−0.405, 1.518)
Age group										
16–34	Ref.		Ref.		Ref.		Ref.		Ref.	
35–54	0.083	(−0.178, 0.387)	0.128	(−0.098, 0.429)	0.093	(−0.206, 0.487)	0.117	(−0.159, 0.444)	0.180	(−0.426, 2.037)
55–74	0.246	(−0.014, 0.613)	0.184	(−0.051, 0.506)	0.178	(−0.107, 0.631)	0.158	(−0.146, 0.513)	0.254	(−0.314, 2.475)
>75	0.146	(−0.148, 0.723)	0.031	(−0.330, 0.451)	0.126	(−0.222, 0.805)	0.099	(−0.266, 0.631)	0.099	(−1.224, 2.475)
Civil status										
Unmarried	Ref.		Ref.		Ref.		Ref.		Ref.	
Divorced/widowed	0.003	(−0.378, 0.388)	−0.011	(−0.348, 0.310)	−0.090	(−0.638, 0.258)	−0.008	(−0.423, 0.394)	0.028	(−1.549, 1.938)
Married/Partnership	0.038	(−0.214, 0.311)	−0.098	(−0.361, 0.107)	−0.090	(−0.465, 0.186)	−0.124	(−0.441, 0.130)	−0.094	(−1.617, 0.745)
Education										
Primary/secondary education	Ref.		Ref.		Ref.		Ref.		Ref.	
Short-cycle higher education	0.021	(−0.336, 0.397)	0.043	(−0.259, 0.381)	**0.264**	(0.012, 0.852)	0.102	(−0.252, 0.521)	0.106	(−1.105, 2.129)
Bachelor’s degree	−0.01	(−0.356, 0.332)	−0.061	(−0.376, 0.226)	0.184	(−0.131, 0.677)	0.069	(−0.288, 0.450)	0.122	(−1.033, 2.081)
Master’s degree or higher	−0.06	(−0.475, 0.284)	−0.124	(−0.571, 0.123)	0.021	(−0.411, 0.503)	−0.069	(−0.536, 0.299)	−0.032	(−1.913, 1.531)
Other	0.053	(−0.285, 0.468)	−0.043	(−0.418, 0.258)	0.010	(−0.424, 0.467)	−0.091	(−0.530, 0.244)	−0.080	(−2.133, 1.184)
Occupational status										
Employed (0)	Ref.		Ref.		Ref.		Ref.		Ref.	
Not employed (1)	0.063	(−0.153, 0.302)	0.006	(−0.187, 0.202)	−0.068	(−0.357, 0.163)	0.111	(−0.110, 0.361)	0.086	(−0.626, 1.339)
Usage of Internet										
Rare or absent (0)	Ref.		Ref.		Ref.		Ref.		Ref.	
Frequent (1)	0.163	(−0.075, 1.184)	0.147	(−0.048, 0.982)	**0.201**	(0.062, 1.481)	−0.005	(−0.743, 0.706)	0.164	(−0.978, 5.181)
IT competence										
Poor (0)	Ref.		Ref.		Ref.		Ref.		Ref.	
Good (1)	0.051	(−0.239, 0.413)	0.130	(−0.062, 0.524)	−0.053	(−0.489, 0.271)	0.052	(−0.267, 0.448)	−0.104	(−2.465, 0.948)
Self-assessed health										
Poor (0)	Ref.		Ref.		Ref.		Ref.		Ref.	
Good (1)	0.109	(−0.087, 0.368)	0.013	(−0.177, 0.213)	0.139	(−0.04, 0.489)	0.079	(−0.136, 0.330)	0.063	(−0.709, 1.302)

Values in bold are statistically significant. * Sample size for the different analyses diverges due to varying numbers of valid answers. The answering option of “don’t know” have been exempted from the analysis.

## 4. Discussion

The findings of this study offer important insights into how patients in Denmark interact with written appointment letters, particularly regarding their understanding of general health information and specific patient rights. One of the key findings was that patients demonstrated relatively strong abilities in understanding and applying basic information from appointment letters, with mean scores of 3.250 and 3.144, respectively, on a 4-point scale.

These results align with previous research which indicates that patients are generally more comfortable with straightforward, concrete information such as appointment dates, times, and locations [17,30]. However, the study also highlights a concerning gap in patients’ ability to assess and appraise more complex information, particularly regarding their rights as patients.

One of the most notable findings was the relatively low mean score (3.070) for the appraising domain, which specifically covers patients’ abilities to evaluate and act upon their patient rights as outlined in appointment letters. This result is consistent with previous literature, which suggests that patients often struggle with understanding their rights, even when this information is provided [31,32].

In this study, participants with higher education levels were more adept at appraising legal information, as indicated by the significant positive association between short-cycle higher education and appraising skills. This finding underscores the critical role of education in enhancing health literacy, particularly in areas that require greater critical thinking and evaluative skills [24].

Moreover, the positive relationship between frequent Internet use and improved appraisal of patient rights highlights the role of digital literacy in navigating healthcare communications. Frequent Internet users may be more likely to encounter not only medical information but also resources on healthcare policies, legal frameworks, and patients’ rights. When combined with eHealth literacy skills, such as the ability to identify credible sources, critically evaluate information, and apply it to one’s own situation, this exposure may translate into a greater capacity to comprehend rights related to healthcare. Thus, eHealth literacy functions as a bridge between digital information environments and patients’ understanding of their entitlements within the healthcare system [33].

This finding aligns with previous research [33] emphasizing the importance of eHealth literacy in improving patient engagement with healthcare resources. However, it highlights a potential digital divide, where patients with limited access to or proficiency with digital tools may be disadvantaged in understanding their rights as patients. Research has shown that patient engagement in healthcare decision-making is significantly influenced by their level of health literacy [34].

Despite these significant findings, the study also revealed that sociodemographic factors such as age, civil status, and employment status did not significantly correlate with health literacy across the assessed domains. This result suggests that, in the Danish healthcare context, these factors may not strongly influence how patients interact with written healthcare communication. However, given the relatively high levels of education and Internet use among the participants, the findings may not be generalizable to populations with lower education levels or limited digital access.

While this pilot study provides evidence that higher education and digital literacy can improve comprehension, future research is needed to determining how best to communicate patient rights in written and digital formats [35]. Moreover, the finding that many patients still struggle to appraise this information suggests that the language used in appointment letters may need to be simplified, or alternative formats, such as visual aids or interactive digital tools, should be considered to help patients better understand their rights.

This study has several limitations that should be acknowledged:

Sample representation: The participant pool primarily consisted of patients from three Danish hospitals, which may limit the generalizability of the findings to broader populations. Because one of the participating departments (Gynecology) serves only women, the overall sample included a higher proportion of female participants. Although sex was included as a variable in the multiple linear regression analysis to account for this imbalance, the skew towards female participants, together with the relatively high educational attainment in the sample, may not accurately reflect the health literacy challenges faced by more diverse populations or those from lower socioeconomic groups.

Sample size: This study did not include a formal sample size calculation and relied on convenience sampling. While this approach is acceptable for a pilot study aimed at exploring feasibility and generating preliminary insights, it does limit the representativeness of the sample and reduces the generalizability of the findings. The results should therefore be interpreted with caution and primarily seen as a basis for informing the design of larger, adequately powered studies.

Self-reported Data: The reliance on self-administered questionnaires introduces potential biases, including social desirability bias, which may lead participants to overestimate their health literacy skills. Additionally, self-assessed health literacy may not align with objective measures, leading to discrepancies between reported understanding and actual comprehension.

A substantial portion of participants (approximately 41%) selected ‘don’t know’ for at least one health literacy item and were excluded from the analysis. We recognize that responding ‘don’t know’ may indicate difficulty with or lack of understanding of the material, suggesting that these responses are not missing at random. Consequently, our complete-case analysis could bias results toward overestimating patients’ health literacy. We therefore caution that the comprehension levels might be lower than our observed averages.

We considered advanced approaches (e.g., multiple imputation) to address the high rate of ‘don’t know’ responses. However, imputing these values could introduce bias, as ‘don’t know’ likely reflects genuinely lower understanding rather than random omissions. We therefore opted to present the complete-case results with caution. Identifying comprehension gaps was one of the aims of this exploratory pilot study. We noted that future larger-scale studies should incorporate techniques such as multiple imputation to evaluate the impact of this nonrandom missingness.

Cross-sectional design: The study’s cross-sectional nature limits its ability to establish causal relationships between sociodemographic factors and health literacy outcomes. Longitudinal studies would be beneficial for understanding how health literacy evolves and its implications for patient rights and engagement.

Limited exploration of patient rights: While the study aimed to assess understanding of patient rights articulated in appointment letters, it did not examine specific patient legal terminologies or concepts that might further complicate patient comprehension. Future research should consider qualitative methods to explore how patients interpret legal rights in healthcare settings.

Digital literacy variability: Although the study acknowledged the role of digital literacy, it did not comprehensively measure participants’ proficiency with digital tools or evaluate their impact on understanding written communications. Given the increasing reliance on digital formats in healthcare, this aspect warrants further investigation.

Addressing these limitations in future research will support a more comprehensive understanding of health literacy and its implications for patient rights.

## 5. Conclusions

The findings from this pilot study suggest there is a concerning gap in patients’ ability to appraise and understand their rights within written healthcare communications. While patients demonstrated reasonable understanding of basic information, critical legal aspects remained challenging, which may indicate a potential need for enhanced education and digital literacy. Furthermore, the role of digital tools in improving health literacy warrants further investigation, particularly as healthcare systems increasingly adopt these technologies. Given the exploratory nature and limitations of this study, these results should be interpreted with caution, but they may provide preliminary directions for future research. Larger, more representative studies are warranted to confirm these findings, to optimize communication strategies for patient rights, and to explore how digital tools can enhance health literacy.

The findings of this study should be considered exploratory and may have preliminary implications for both policy and practical applications in healthcare. Rather than recommending immediate implementation, we suggest that healthcare providers could explore tailored communication methods that account for varying levels of health literacy among patients. Approaches such as teach-back techniques and providing educational materials in multiple formats (e.g., plain language with low LIX scores and visual aids) could be evaluated in future studies to determine their effectiveness in empowering patients to better understand their rights and responsibilities. Similarly, healthcare policies might benefit from future investigation into whether mandating simplified language in written communications, particularly regarding patients’ legal rights, improves comprehension at the population level. Policymakers could also consider piloting health literacy training within healthcare provider education to strengthen communication strategies.

## Data Availability

The fully anonymized dataset that support the findings of this study are available on reasonable request from the corresponding author. The data are not publicly available due to privacy/ethical restrictions.

## Supplementary Materials

The following supporting information can be downloaded at: https://www.mdpi.com/article/10.3390/ijerph22101518/s1, Supplementary Material S1: Example of Danish pre-appointment letter; Figure S1: Questionnaire of health literacy in written appointment letters, in Danish; Figure S2: Analytical framework; Table S1: Multiple linear regressions on factors associated with self-assessed health literacy domains of assessing, understanding, appraising, and applying written appointment letters—crude model 1.
