# Supplementary material for: Understanding Patient Rights: A Pilot Study Assessing Health Literacy in Written Pre-Appointment Letters
Source: Int J Environ Res Public Health. 2025 Oct 3;22(10):1518. doi: 10.3390/ijerph22101518 (PMC12562880; doi:10.3390/ijerph22101518)
Supplement: Supplementary file 1 [file ijerph-22-01518-s001.zip › ijerph-3847252-supplementary.pdf]

Understanding Patient Rights: A Pilot study Assessing Health Literacy in Written Pre-appointment Letters

Supplementary materials

Supplementary material S1:

Example of Danish pre-appointment letter

Hospital A

City X
Address Y
Orthopedic Outpatient Clinic

Date: [Date, year]

[Patient name and address],

We have received your referral on [Referral Date], and have given you an appointment for a pre-examination regarding [xxx]:

[Date, time]
Department
Adress
Use entrance [Address] or [Address]. Follow the main corridor to section B.

Your rights as a patient

You have the right to be examined and have your condition clarified (the right to examination) within 30 days from the date we received your referral.

We cannot offer this to you in this department, but it can be done at Hospital X, City x.

If you would like to be examined there, you must call us as soon as possible, and we will forward your referral. Our phone number is shown in the upper right corner.

You can read more about your rights on the next page.

What you need to do before
Be aware that you may be called in for an X-ray before your appointment

You must complete the health declaration which is attached as an appendix. Press “submit” to send the health declaration electronically.

This is how it works
At the [webpage] you can read more about the department.

If surgery is agreed upon, a medical record will be made, possibly supplemented by blood tests. You must also speak with an anesthesiologist or anesthesia nurse. This can take place either as an in-person meeting or via video consultation.

What to do when you arrive
On [webpage] you can find the map of the hospital and read about parking.

Scan your health card at the self-service terminal and follow the on-screen instructions.

Contact us
If you are prevented from attending, you must contact us as soon as possible at Phone [xxxx], Monday–Friday, 08:30–13:30.

Kind regards,
X Clinic

Your rights as a patient
You have the right to be examined and have your condition clarified, and that must happen within 30 days from when we received your referral.

We cannot meet this right in our department, but you have the following options:

- You can **keep the appointment** we have given you.
  - You don't need to do anything other than show up at the time mentioned at page 1. Be aware that there may be things you must prepare before your visit to the hospital. See page 1.

- You can also **be examined at Hospital, mentioned at page 1**
  - You must call us, because then we will forward your referral.
  The phone number is at the top right corner on page 1.

- You can also **choose another public hospital in Denmark.** This right is called "the free hospital choice" and applies to all patients.
  At [webpage] you can see waiting times at other hospitals.
  - You must call us if you wish to be examined or treated at another hospital, because then we will forward your referral.
  The phone number is at the top right corner on page 1.

Other information WORTH KNOWING
Your rights are given by the Health Act. You can read more on [webpage]. If you do not have internet access, you can call us and we will send you the leaflet *"When you are referred to hospital"*, which tells you about your rights.
The phone number is at the top right corner on page 1.

- about the number of treatments
You have the right to know how many treatments we carry out of the type you are waiting for. Call us. The phone number is at the top right corner on page 1.

- about transport
As a rule, you must arrange your own transport to the hospital. For health reasons, you may be entitled to hospital transport or financial support. Read more on [webpage] or call the Transport Office at [phone number].

- about personal data
We collect different relevant personal data about you as a patient. This information may come from your general practitioner. You have the right to know how we collect and process such information. Read more on [webpage].

- further information
You may contact a patient advisor at the Patient Office at [phone number], if you need more information about your rights, waiting times, or the free hospital choice.

If you live outside Region X, you must contact the Patient Office in your own region.

Supplementary material S2:

På en skala fra meget let til meget svært, hvor let vil du så sige, det er at:	
Q1:	...finde information om behandling af sygdomme de vedrører dig??
Q2:	...finde ud af hvor du kan finde professionel hjælp (læge, apotek mm)?
Q3:	...finde en støtteperson (partner, ægtefælle, ven, ledsagehjælp) i forhold til at få støtte til at orientere dig i sundhedssystemet?
Q4:	...finde informationer om dine rettigheder som patient (f.eks. din rettighed til at blive udredt inden for 30 dage)?
Q5:	…få overblik over de informationer der er udsendt til dig ifm. din forundersøgelse?
Q6:	…forstå indkaldelsesbrevet (siden hvor dato og klokkeslæt kan ses) der er udsendt til dig i forbindelse med din forundersøgelse?
Q7:	…forstå de bilag der er vedhæftet din indkaldelse til forundersøgelse, herunder specifikt ift. dine rettigheder som patient (f.eks. din rettighed til at blive udredt inden for 30 dage?)
Q8:	…vurdere om du har rettigheder du kan anvende (f.eks. din rettighed til at blive udredt inden for 30 dage)?
Q9:	…vurdere om du har brug for støtteperson (partner, ægtefælle, ven, ledsagehjælper) til råd og vejledning/hjælp ift. at forstå og få overblik over de informationer der er sendt til dig?
Q10:	…bruge den information, som er vedhæftet som bilag dit indkaldelsesbrev, til at træffe beslutninger om dine rettigheder og muligheder?
Q11:	…at forberede spørgsmål til konsultation/forundersøgelsen?
Q12:	…bruge den information, du har modtaget ifm. med din forundersøgelse, til at træffe beslutninger om din sygdom?
Q13:	…følge vejledning fra lægen du mødte til forundersøgelsen?

Figure S1: Questionnaire of health literacy in written appointment letters, in Danish

Supplementary material S3:

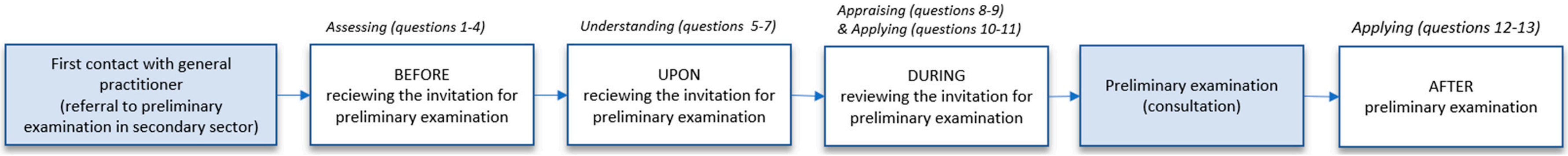

Figure S2: Analytical framework*
* Each health-literacy domain (assessing, understanding, appraising, and applying) operationalizes *patient rights comprehension* in appointment letters. Specifically, items that explicitly reference rights are distributed across domains as follows: Q4 (assessing—*find information about your rights*), Q7 (understanding—*understand attachments regarding rights*), Q8 (appraising—*judge whether a right applies*), and **Q10** (applying—*use the letter to decide your rights and options*). The remaining items capture generic processing steps (locating providers/support, understanding logistics, preparing questions, acting on information) that enable patients to exercise rights in practice.

Supplementary material S4: **Table S1:** Multiple linear regressions on factors associated with self-assessed health literacy domains of assessing, understanding, appraising, and applying written appointment letters – crude model 1

	**Assessing**		**Understanding**		**Appraising**		**Applying**		**Health Literacy Total**	
	Crude - model 1 n = 237*		Crude - model 1 n = 315*		Crude - model 1 n = 257*		Crude - model 1 n = 213*		Crude - model 1 n = 164*	
	β	95% CI	β	95% CI	β	95% CI	β	95% CI	β	95% CI
**Sex**										
Male (0)	Ref.		Ref.		Ref.		Ref.		Ref.	
Female (1)	0.043	(-0.118, 0.235)	**0.142**	(0.044, 0.355)	-0.036	(-0.258, 0.140)	0.067	(-0.096, 0.284)	0.098	(-0.279, 1.247)
**Age group**										
16 - 34	Ref.		Ref.		Ref.		Ref.		Ref.	
35 - 54	-0.040	(-0.260, 0.163)	0.076	(-0.106, 0.298)	0.102	(-0.160, 0.395)	0.010	(-0.220, 0.245)	0.070	(-0.609, 1.231)
55 - 74	0.078	(-0.112, 0.292)	0.018	(-0.173, 0.216)	0.152	(-0.036, 0.447)	0.049	(-0.165, 0.280)	0.141	(-0.286, 1.452)
> 75	0.037	(-0.230, 0.384)	-0.061	(-0.399, 0.147)	0.101	(-0.107, 0.569)	-0.009	(-0.335, 0.298)	0.002	(-1.324, 1.349)
**Civil status**										
Unmarried	Ref.		Ref.		Ref.		Ref.		Ref.	
Divorced/widowed	0.040	(-0.200, 0.337)	-0.033	(-0.283, 0.176)	0.020	(-0.252, 0.325)	-0.026	(-0.331, 0.244)	0.064	(-0.834, 1.711)
Married/Partnership	0.066	(-0.110, 0.271)	-0.038	(-0.224, 0.130)	0.101	(-0.085, 0.373)	-0.086	(-0.320, 0.104)	0.004	(-0.835, 0.872)
**Education**										
Primary/secondary education	Ref.		Ref.		Ref.		Ref.		Ref.	
Short-cycle higher education	0.006	(-0.259, 0.276)	0.095	(-0.100, 0.371)	0.125	(-0.091, 0.493)	0.059	(-0.214, 0.382)	0.058	(-0.916, 1.525)
Bachelor's degree	0.072	(-0.154, 0.320)	0.043	(-0.163, 0.267)	0.113	(-0.114, 0.424)	0.070	(-0.191, 0.359)	0.132	(-0.544, 1,666)
Master's degree or higher	-0.024	(-0.314, 0.241)	-0.019	(-0.304, 0.234)	0.007	(-0.317, 0.348)	-0.016	(-0.352, 0.300)	0.021	(-1.146, 1.380)
Other	0.079	(-0.157, 0.418)	0.037	(-0.201, 0.334)	-0.023	(-0.372, 0.283)	0.006	(-0.302, 0.320)	0.002	(-1.275, 1.294)
**Occupational status**										
Employed (0)	Ref.		Ref.		Ref.		Ref.		Ref.	
Not employed (1)	0.021	(-0.123, 0.171)	0.093	(-0.022, 0.242)	0.031	(-0.124, 0.207)	**0.138**	(0.003, 0.316)	0.150	(-0.204, 1.070)
**Usage of Internet**										
Rare or absent (0)	Ref.		Ref.		Ref.		Ref.		Ref.	
Frequent (1)	**0.139**	(0.036, 0.838)	**0.141**	(0.091, 0.747)	0.097	(-0.088, 0.759)	-0.028	(-0.470, 0.307)	0.083	(-0.786, 2.584)
**IT competence**										
Poor (0)	Ref.		Ref.		Ref.		Ref.		Ref.	
Good (1)	**0.130**	(0.005, 0.404)	**0.180**	(0.112, 0.461)	0.054	(-0.126, 0.322)	0.058	(-0.128, 0.319)	0.047	(-0.707, 1.322)
**Self-assessed health**										
Poor (0)	Ref.		Ref.		Ref.		Ref.		Ref.	
Good (1)	0.116	(-0.046, 0.352)	0.028	(-0.140, 0.215)	0.063	(-0.140, 0.343)	0.092	(-0.087, 0.314)	0.042	(-0.669, 1.061)

*Sample size for the different analyses diverges due to varying numbers of valid answers. The answering option of "don't know" have been exempted from the analysis.
